# Clinical characteristics and long-term prognosis of anti-MDA5-positive dermatomyositis: a comparative study across age groups

**DOI:** 10.1186/s13023-026-04345-y

**Published:** 2026-04-11

**Authors:** Fang Dong, Panpan Zhang, Wenlu Hu, Rui Liu, Tianqi Li, Wenhui Lou, Jinlei Sun, Yujie He, Shengyun Liu, Yusheng Zhang

**Affiliations:** Clinical Immunology and Rheumatology, The First Affiliated Hospital of Zhengzhou University, Zhengzhou University, Zhengzhou, Henan Province China

**Keywords:** Anti-melanoma differentiation-associated gene 5, Different age groups, Clinical manifestations, Long-term prognosis, Nomogram model

## Abstract

**Objectives:**

Research focused on clinical differences and long-term prognosis in anti-melanoma differentiation-associated gene 5 antibody-positive dermatomyositis (anti-MDA5+ DM) patients across age groups remains limited. This study aimed to explore the differences in the clinical manifestations and long-term mortality of anti-MDA5+ DM patients across age groups.

**Methods:**

We included 318 newly diagnosed anti-MDA5+ DM patients, recruited from June 2018 to January 2024. The median follow-up time was 22.5 months (4.5–36 months). The Cochran-Armitage test for trend (CATT) was employed to assess the statistical significance of changes in the proportion of clinical characteristics across different age groups. Cox regression analysis and a nomogram model were developed to stratify the risk associated with mortality.

**Results:**

In the cohort of 318 patients, 123 (38.7%) were aged < 50 years, 124 (39.0%) were aged 50–59 years, and 71 (22.3%) were aged ≥ 60 years. Clinical manifestations and comorbidities such as cough, *Pneumocystis jirovecii* pneumonia (PJP), dyspnea, and rapidly progressive interstitial lung disease (RP-ILD) increased with age, while rash and arthralgia decreased. PJP was a major factor in poor prognosis, especially among older patients who were more susceptible to infection. The nomogram, the first prognostic model incorporating both age and PJP infection in anti-MDA5+ DM, demonstrated its independent and combined effects on mortality and enabled early risk stratification, providing a valuable tool for clinical decision-making.

**Conclusions:**

Clinical manifestations and laboratory parameters varied in anti-MDA5+ DM patients across different age groups. Advanced age and PJP are major factors associated with poor prognosis, with patients aged ≥ 60 years showing the highest mortality and being predominat in the high-risk group.

**Supplementary Information:**

The online version contains supplementary material available at 10.1186/s13023-026-04345-y.

## Introduction

Anti-melanoma differentiation-associated gene 5 antibody-positive dermatomyositis (anti-MDA5+ DM) is a rare but distinct subtype of idiopathic inflammatory myopathy (IIM), predominantly reported in East Asia [1, 2]. The anti-MDA5 antibody was initially identified by Sato et al. in 2005 and was officially designated as such in 2009 [3, 4]. Patients with anti-MDA5+ DM generally exhibit minimal or no overt muscle involvement; however, they are more susceptible to developing rapidly progressive interstitial lung disease (RP-ILD) [5]. Research has demonstrated that the presence of anti-MDA5 antibody is an independent predictor of reduced survival rates [6, 7], thereby presenting significant challenges in treatment and contributing to increased mortality rates among affected patients.

With the aging of the population, older patients account for an increasing proportion of autoimmune diseases, and the clinical features as well as the prognosis of many autoimmune diseases are age-related [8]. In patients with rheumatoid arthritis (RA), the prevalence of the disease shows an overall increasing trend with age [9]. In contrast, it has been shown that patients with late-onset RA (LORA) are more prone to an increase in acute-phase reactants as well as sub(acute) symptomatic episodes, as well as to a higher degree of disease activity and a poorer prognosis [10–12]. Regarding systemic lupus erythematosus (SLE) patients, arthritis, serositis, and peripheral neuropathy are more likely to be present with increasing age, while alopecia, lupus nephritis, and neuropsychiatric SLE (NPSLE) become less common. Additionally, late-onset SLE patients have a relatively lower 10-year survival rate [13, 14]. In Sjögren’s syndrome (SS), the prevalence of dryness and interstitial lung disease (ILD) tends to increase with the age of onset [15].

Similar age-related variations are observed in anti-MDA5+ DM, with studies reporting that older patients have worse clinical outcomes and advanced age is an independent risk factor for mortality [16, 17]. However, these studies provide only partial insights into the association between age and clinical course. Comprehensive analyses across a full age spectrum are lacking.

Current literature includes only limited comparisons of clinical features between older and younger anti-MDA5+ DM patients. For example, Yamaguchi et al. [18] analyzed clinical outcomes in 40 patients with anti-MDA5+ DM and reported significantly poorer prognosis in those aged ≥ 60 years. He & Zhou et al. [19] enrolled 251 anti-MDA5+ DM patients and found that mortality risk increased notably in patients over 54 years. Nonetheless, large-scale studies evaluating age-related differences in clinical features, laboratory examinations, and long-term prognosis remain scarce. To address this gap, we analyzed 318 anti-MDA5+ DM patients stratified by age to investigate differences in clinical manifestations, comorbidities, laboratory examinations, treatment regimens, and outcomes. In addition, we developed a risk stratification model to support earlier diagnosis, targeted interventions, and personalized follow-up. For high-risk patients, early and intensive treatment is recommended to improve survival and reduce disease burden.

## Methods

### Patients’ enrollment

A total of 318 inpatients at the First Affiliated Hospital of Zhengzhou University, from June 2018 to January 2024, were retrospectively recruited for this study. Among them, 84 patients died within the first 6 months, and 4 patients were lost to follow-up. The median follow-up duration was 22.5 months (range: 4.5–36 months). A minimum follow-up period of 6 months was required for the remaining patients, given that numerous studies indicate that mortality predominantly occurs within the first 6 months [20, 21]. The inclusion criteria required all participants to test positive for anti-MDA5 antibody and to fulfill either the 2004 European Neuromuscular Centre (ENMC) criteria [22] for DM or the Bohan and Peter criteria [23]. For patients with clinically amyopathic DM (CADM), the diagnosis was primarily based on the ENMC criteria, which incorporate characteristic skin manifestations even in the absence of overt muscle involvement. Exclusion criteria encompassed patients diagnosed with SLE, SS, RA, and other connective tissue diseases, as well as those diagnosed with chronic infectious diseases before admission. All clinical data were sourced from the hospital’s medical record system. This study was approved by the Ethics Committee of the First Affiliated Hospital of Zhengzhou University (2021-KY-1101).

### Data collection

After determining the cut-off value using the Restricted Cubic Spline (RCS) curve [24], and considering the age distribution of the cohort, all patients were categorized into three groups based on their age at diagnosis: <50 years (*n* = 123), 50–59 years (*n* = 124), and ≥ 60 years (*n* = 71). A retrospective analysis was then performed, using baseline data collected from the hospital’s electronic medical record system at the time of diagnosis. This included demographic characteristics, clinical features, laboratory test results, treatment regimens, and prognostic outcomes.

RP-ILD was defined as acute progressive dyspnea and hypoxemia, accompanied by exacerbation of ILD on high-resolution computed tomography (HRCT) within 4 weeks following the onset of respiratory symptoms [3]. Pulmonary function test data included measurements of Forced Vital Capacity (FVC), FVC% pred, Diffusing Capacity of the Lungs for Carbon Monoxide (DLCO), and DLCO% pred. Blood gas analysis data encompassed parameters such as the PaO₂/FiO₂ ratio and the alveolar-arterial oxygen gradient (AaDO₂), calculated using the formula P(A-a)O₂ = (PiO₂ - PaCO₂/R) - PaO₂. Pulmonary function data were often unavailable in patients aged ≥ 60 years, primarily because those with RP-ILD could not undergo pulmonary function testing owing to severe dyspnea and concern for further pulmonary injury.

In our cohort, the diagnosis of *Pneumocystis jirovecii* pneumonia (PJP) was established in patients diagnosed with PJP based on clinical manifestations, HRCT findings, and serum (1,3)-β-D-glucan (BDG) results, and these patients further underwent metagenomic next-generation sequencing (mNGS) of bronchoalveolar lavage fluid (BALF) to confirm the infection. PJP was classified as initial if it occurred before or within 72 h of hospital admission, prior to intensive immunosuppressive therapy, and defined as post-treatment PJP if it developed afterward. For patients with multiple PJP episodes, only the first event was included.

Detection of autoantibodies was performed using standardized commercial kits. Specifically, anti-MDA5 antibodies were measured using an enzyme-linked immunosorbent assay (ELISA) kit (MBL Co., Ltd., Japan), in accordance with the manufacturer’s instructions. Anti-Ro-52 antibodies were identified using an indirect immunofluorescence assay (Euroimmun AG, Germany).

### Treatment regimens and outcome

The initial treatment regimen for patients primarily included high-dose glucocorticoids (GC), cyclophosphamide (CYC), calcineurin inhibitors (CNI, such as tacrolimus or cyclosporine), tofacitinib, and intravenous immunoglobulin (IVIG). High-dose glucocorticoids were defined as 1–2 mg/(kg·d) of prednisone-equivalent. In our cohort, patients received high-dose methylprednisolone (MP), with doses converted to prednisone-equivalent for consistency. The initial therapeutic approach varied and included monotherapy with steroids, dual therapy, triple therapy with immunosuppressive agents, and the potential addition of intravenous immunoglobulin therapy, based on the combination of these drugs. The primary outcome measure was mortality. Survival status was assessed through hospital records or follow-up telephone calls.

### Statistical analysis

Continuous variables were summarized as means ± SD or medians with interquartile ranges, and categorical variables as counts and percentages. Group comparisons were performed using one-way ANOVA or Kruskal-Wallis tests, with post hoc analyses as appropriate. Trends across age groups were assessed with the Cochran-Armitage test. Correlations were evaluated using Pearson or Spearman coefficients. Restricted cubic splines (RCS) were used to model the relationship between age and mortality. Survival analysis utilized Kaplan-Meier curves with log-rank tests. Risk factors were identified via univariate and multivariate Cox regression, with stepwise selection for the final model. A prognostic nomogram was developed using significant Cox variables. Risk groups were defined by X-tile, and model performance was assessed via the C-index, AUC, and Hosmer-Lemeshow test. Analyses were performed with SPSS (v26.0), R (v4.3.1), and X-tile (v3.6.1). A two-sided *P* < 0.05 was considered statistically significant.

## Results

### Comparison of demographic features across age groups

**Fig. 1:**
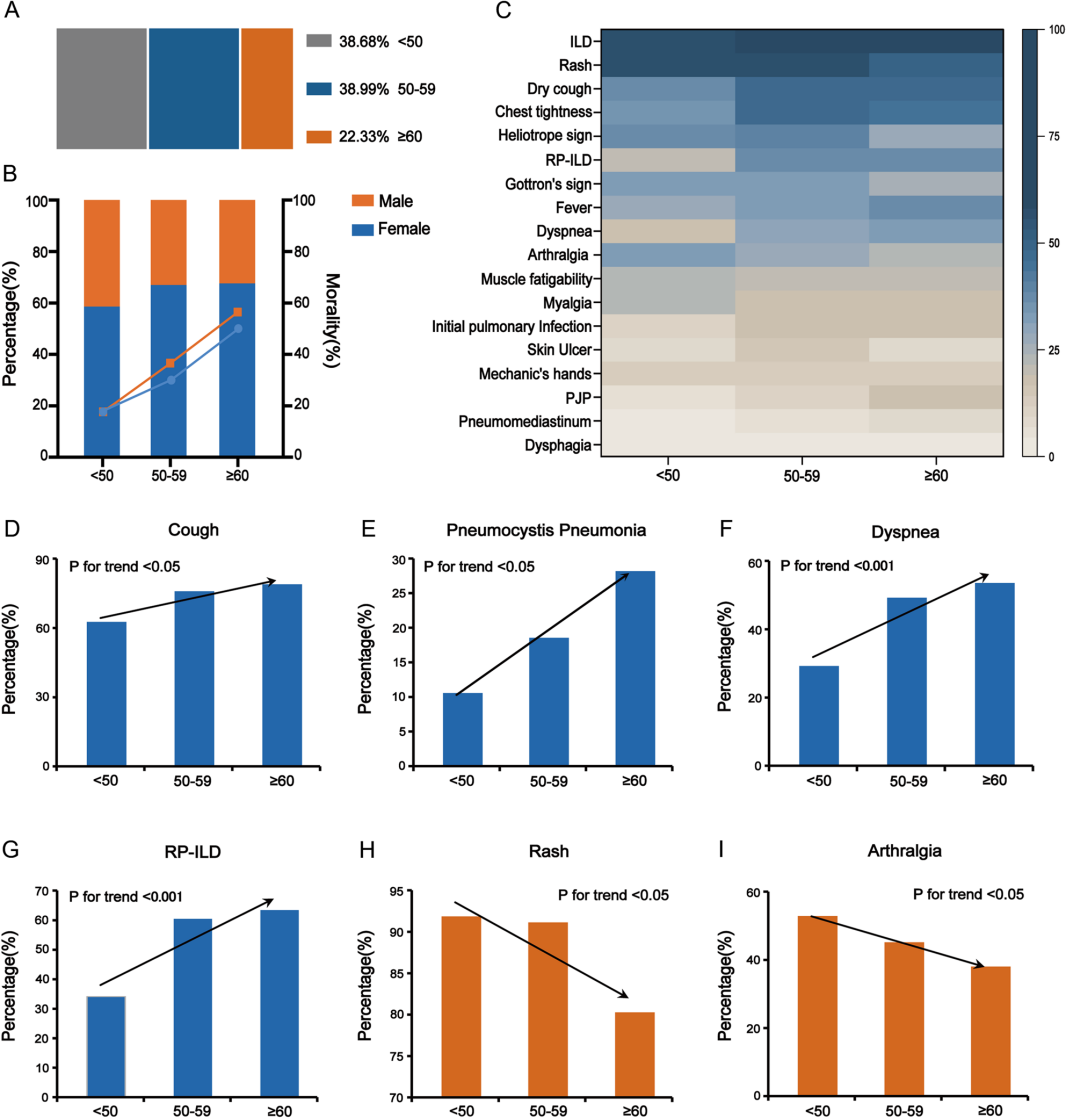
Clinical and demographic characteristics of anti-MDA5-positive dermatomyositis across age groups. **(A)** Distribution of anti-MDA5+ DM patients across age groups. **(B)** Sex ratio and mortality rates across different age groups. **(C)** Heatmap of organ involvement frequency (%) across age groups. **(D-I)** Comparison of the trends in clinical manifestations: **(D)** Cough, **(E)** PJP, **(F)** Dyspnea, **(G)** RP-ILD, **(H)** Rash, **(I)** Arthralgia

In this study, a total of 318 patients with anti-MDA5+ DM were included and categorized into three age groups based on their age at diagnosis (Fig. 1A). The demographic characteristics of these patients are detailed in Table 1. Gender distribution and mortality rates were compared across the three age groups (Fig. 1B). Our findings indicate that females accounted for a higher proportion in each age group compared to males.

**Table 1:** Baseline demographic manifestations and clinical features across age groups

Variables	< 50 (*n* = 123)	50–59 (*n* = 124)	≥ 60 (*n* = 71)	*P*
**Demographic features**				
Age (years), M (Q₁, Q₃)	42(36,47)	55(52,57)	66(63,69)	**< 0.001**
Male, n (%)	51 (41.5)	41 (33.1)	23 (32.4)	0.294
Disease duration (months), M (Q₁, Q₃)	2.1 (1.1,3.5)	1.6 (1.1,3.2)	1.8(1.1,3.2)	0.516
Smoking, n (%)	14 (11.4)	14 (11.3)	9 (12.7)	0.953
Diabetes, n (%)	27 (22.0)	38 (30.7)	26 (36.6)	0.076
**Clinical features**				
ILD, n (%)	114 (92.7)	118 (95.2)	70 (98.6)	0.192
RP-ILD, n (%)	42 (34.2)	75 (60.5)	45 (63.4)	**< 0.001**
Rash, n (%)	113 (91.9)	113 (91.1)	57 (80.3)	**0.028**
Skin ulcer, n (%)	18 (14.6)	31 (25.0)	9 (12.7)	**0.042**
Mechanic’s hands, n (%)	26 (21.1)	29 (23.4)	15 (21.1)	0.894
Heliotrope sign, n (%)	77 (62.6)	82 (66.1)	33 (46.5)	**0.021**
Gottron’s sign, n (%)	66 (53.7)	69 (55.7)	29 (40.9)	0.116
Myalgia, n (%)	46 (37.4)	37 (29.8)	22 (31.0)	0.413
Muscle fatigability, n (%)	42 (34.2)	36 (29.0)	25 (35.2)	0.586
Cough, n (%)	77 (62.6)	94 (75.8)	56 (78.9)	**0.020**
Chest tightness, n (%)	73 (59.4)	94 (75.8)	52 (73.2)	**0.013**
Dyspnea, n (%)	36 (29.3)	61 (49.2)	38 (53.5)	**< 0.001**
Arthralgia, n (%)	65 (52.9)	56 (45.2)	27 (38.0)	0.127

Disease duration, the time from symptom onset to diagnosis; ILD, interstitial lung disease; RP-ILD, rapidly progressive interstitial lung disease

### Comparison of clinical manifestations and comorbidities across age groups

To elucidate and visualize the clinical manifestations and comorbidities of anti-MDA5+ DM across different age groups, a heat map was generated (Fig. 1C). This visualization highlighted the varying frequencies of clinical features among the age groups. Notably, there were significant differences across age groups in the prevalence of skin ulcers (*P* = 0.042), heliotrope signs (*P* = 0.021), cough (*P* = 0.020), chest tightness (*P* = 0.013), and RP-ILD (*P* < 0.001). However, there were no significant differences across age groups in the prevalence of Mechanic’s hands, Gottron’s sign, myalgia, or muscle fatigability.

The comorbidities, respiratory assessments, treatment regimens, and outcomes across different age groups are summarized in Table 2. Regarding complications and respiratory assessments, no significant differences were observed among the three age groups in the incidence of pneumomediastinum (*P* = 0.451), initial *Pneumocystis jirovecii* pneumonia (i-PJP) (*P* = 0.077), or post-treatment PJP (pt-PJP) (*P* = 0.139). However, the overall prevalence of PJP, including both initial and post-treatment episodes, increased significantly with age (*P* = 0.008), rising from 10.6% in patients aged < 50 years to 28.2% in those aged ≥ 60 years.

**Table 2:** Baseline comorbidities, respiratory assessment, treatment regimens, and outcome across age groups

Variables	< 50 (*n* = 123)	50–59 (*n* = 124)	≥ 60 (*n* = 71)	*P*
**Complications**				
Pneumomediastinum, n (%)	9 (7.3)	13 (10.5)	9 (12.7)	0.451
*Pneumocystis jirovecii* pneumonia, n (%)	13 (10.6)	23 (18.6)	20 (28.2)	**0.008**
i-PJP, n (%)	7 (5.7)	14 (11.3)	11 (15.5)	0.077
pt-PJP, n (%)	6 (4.9)	9 (7.3)	9 (12.7)	0.139
**Pulmonary function** ^**1**^				
FVC%pred, M (Q₁, Q₃)	70.2 (58.9,83.4)	73.0 (58.1,87.3)	NA	0.110
FVC, L, M (Q₁, Q₃)	2.3 (1.9,3.0)	2.3 (1.9,2.7)	NA	0.367
DLCO, ml/min/mmHg, M (Q₁, Q₃)	4.7 (3.5,5.8)	4.1 (3.3,5.2)	NA	0.051
DLCO%pred, M (Q₁, Q₃)	54.2 (46.1,66.0)	52.3 (42.4,63.6)	NA	0.359
**Arterial blood gas test**				
PaO_2_/FiO_2_(mmHg), M (Q₁, Q₃)	303.6(216.4,370.2)	294.5(205.6,358.1)	204.6(115.2,327.1)	**< 0.001**
AaDO_2_(mmHg), M (Q₁, Q₃)	43.90(22.2,113.2)	50.34(28.1,121.6)	119.2(33.5,333.6)	**< 0.001**
**Treatment regimens**				
Dosage of MP (mg/d), M (Q₁, Q₃)	80 (80,80)	80 (80,80)	80(40,80)	**0.019**
GC mono, n (%)	7 (5.7)	10 (8.1)	13 (18.3)	**0.012**
**Dual therapy**,** n (%)**				
MP + CNI, n (%)	30 (24.4)	29 (23.4)	21 (29.6)	0.612
MP + CYC, n (%)	12 (9.8)	20 (16.1)	12 (16.9)	0.244
MP + JAKi, n (%)	12 (9.8)	4 (3.2)	1 (1.4)	**0.018**
**Triple therapy**^**2**^, **n (%)**				
MP + CNI + CYC, n (%)	35 (28.5)	33 (26.6)	17 (24.0)	0.791
MP + CNI + JAKi, n (%)	24 (19.5)	26 (21.0)	7 (9.9)	0.127
**Add-on therapy**,** n (%)**				
RTX, n (%)	1 (0.8)	1 (0.8)	0 (0.0)	1.000
IVIG, n (%)	74 (60.2)	89 (71.8)	52 (73.2)	0.077
**Outcome**				
Follow-up duration	25.7 (12.5,36.0)	23.4 (3.7,36.0)	8.4 (2.2,33.3)	**< 0.001**
Deaths within 3 years, n (%)	22 (17.9)	40 (32.3)	37 (52.1)	**< 0.001**
Female, n (%)	13 (18.1)	25 (30.1)	24 (50.0)	**< 0.001**
Male, n (%)	9 (17.6)	15 (36.6)	13 (56.5)	**0.003**

PJP, *pneumocystis jirovecii* pneumonia; i-PJP, initial PJP; pt-PJP, post-treatment PJP; FVC, forced vital capacity; DLCO, diffusing capacity for carbon monoxide; AaDO_2,_ arterial-alveolar oxygen gradient, P(A-a)O_2_=(PiO_2_-PaCO_2_/R)-PaO_2_; GC mono, only glucocorticoids were administered initially; MP, methylprednisolone; CNI, calcineurin inhibitors; CYC, cyclophosphamide; JAKi, janus kinase inhibitors; RTX, rituximab; IVIG, intravenous immunoglobulin

^1^Pulmonary function tests were often missing in patients ≥ 60 y due to RP-ILD, test intolerance, and risk of further lung injury

^2^Triple therapy refers to glucocorticoids combined with any two immunosuppressants. In our cohort, the most commonly employed regimens were MP + CNI + CYC or MP + CNI + JAKi

In terms of pulmonary function, no significant differences were found among age groups in FVC% pred (*P* = 0.110), absolute FVC values (*P* = 0.367), DLCO (mL/min/mmHg) (*P* = 0.051), or DLCO% pred (*P* = 0.359). However, arterial blood gas analyses revealed a significantly lower PaO₂/FiO₂ ratio and higher AaDO₂ in patients aged ≥ 60 years compared to younger groups (both *P* < 0.001), indicating more severe gas exchange impairment in the older population.

Further analysis of age-related differences in clinical manifestations and comorbidities involved comparing trends and patterns across age groups. The incidence of cough (*P for trend* < 0.05), PJP (*P for trend* < 0.05), dyspnea (*P for trend* < 0.001), and RP-ILD (*P for trend* < 0.001) demonstrated significant uptrends with advancing age (Fig. 1D-G). Conversely, the frequency of rash (*P for trend* < 0.05) and arthralgia (*P for trend* < 0.05) significantly decreased with age (Fig. 1H-I).

### Comparison of treatment regimens across age groups

Regarding treatment regimens, no significant differences were observed among the three age groups in the use of dual therapy (glucocorticoids (GC) combined with calcineurin inhibitors (CNI) or cyclophosphamide (CYC)), triple therapy (defined as GC combined with any two immunosuppressants, most commonly GC + CNI + CYC or GC + CNI + JAKi), or intravenous immunoglobulin (IVIG). However, patients aged ≥ 60 years received a lower median GC dose (*P* = 0.019) and used GC monotherapy more frequently (*P* = 0.012) than younger groups. In contrast, GC combined with Janus kinase inhibitors (JAKi) was more common in patients aged < 50 years (9.8%) versus older groups (*P* = 0.018). Rituximab (RTX) use was rare and showed no significant intergroup differences.

During long-term follow-up, 99 patients (31.1%) died. The all-cause mortality within 3 years varied significantly among age groups (*P* < 0.001), with a notable increasing trend in mortality rates with advancing age. Specifically, the mortality rate for patients < 50 years was 17.9%, while for those aged ≥ 60 years, the mortality rate increased to 52.1%. In the < 50 years group, the mortality rate was 17.6% in males and 18.1% in females. In the 50–59 years group, mortality was 36.6% in males and 30.1% in females, while in the ≥ 60 years group, mortality was 56.5% in males and 50.0% in females.

### Distribution of laboratory parameters across age groups

**Fig. 2:**
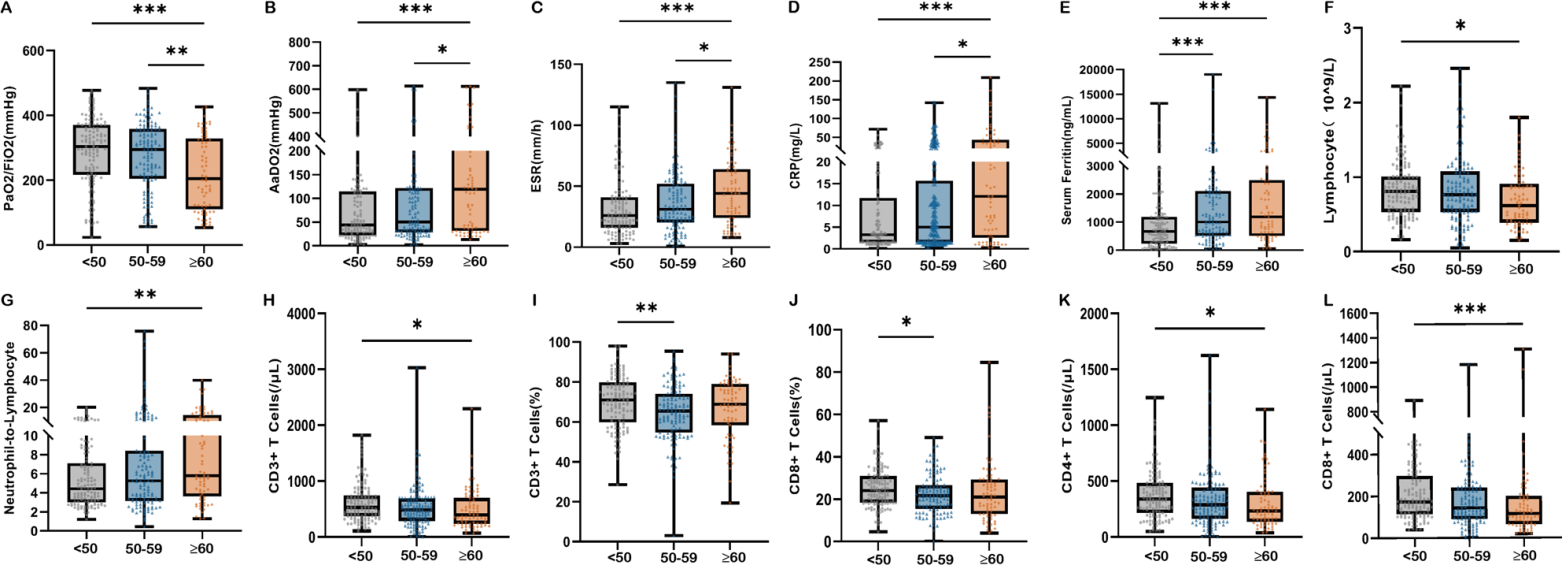
Differences in laboratory examinations across age groups in anti-MDA5-positive dermatomyositis patients. Box-whisker plots (median, IQR) showing: **(A)** PaO₂/FiO₂ ratio, **(B)** AaDO₂, **(C)** Erythrocyte sedimentation rate (ESR), **(D)** C-reactive protein (CRP), **(E)** Serum ferritin, **(F)** Lymphocyte count (LYM), **(G)** Neutrophil-to-lymphocyte ratio (NLR), **(H)** CD3^+^ T cell count, **(I)** CD3^+^ T cell ratio, **(J)** CD4^+^ T cell count, **(K)** CD8^+^ T cell count, **(L)** CD8^+^ T cell ratio. (*p < 0.05, **p < 0.01, ***p < 0.001)

Baseline laboratory examinations were compared across the three age groups (Table 3). Significant differences were observed in several indicators, including lymphocyte count (*P* = 0.022), neutrophil-to-lymphocyte ratio (NLR) (*P* = 0.010), erythrocyte sedimentation rate (ESR) (*P* < 0.001), C-reactive protein (CRP) (*P* < 0.001), percentage of anti-Ro-52+ (*P* < 0.001), CD3^+^ T cell count (*P* = 0.012), CD3^+^ T cell ratio (*P* = 0.008), CD4^+^ T cell count (*P* = 0.011), CD8^+^ T cell count (*P* < 0.001), and CD8^+^ T cell ratio (*P* = 0.019). Further intergroup comparisons of these significant indicators are shown in Fig. 2. Patients aged ≥ 60 years exhibited higher levels of AaDO₂ (*P* < 0.001), ESR (*P* < 0.001), CRP (*P* < 0.001), serum ferritin (*P* < 0.001), and NLR (*P* < 0.01) compared to those aged < 50 years. Conversely, levels of PaO₂/FiO₂ (*P* < 0.001), lymphocyte count (*P* < 0.05), CD3^+^ T cell count (*P* < 0.05), CD4^+^ T cell count (*P* < 0.05), and CD8^+^ T cell count (*P* < 0.001) were significantly lower in the older age group.

**Table 3:** Baseline laboratory examinations across age groups

Variables	< 50 (*n* = 123)	50–59 (*n* = 124)	≥ 60 (*n* = 71)	*P*
Anti-Ro52^+^, n (%)	56 (45.5)	80 (64.5)	51 (71.8)	**< 0.001**
Anti-MDA5 (U/mL), M (Q₁, Q₃)	174.3 (150.9,200.7)	178.5 (155.6,198.5)	181.3 (158.4,209.7)	0.435
LYM (10^9^/L), M (Q₁, Q₃)	0.8 (0.5,1.0)	0.8 (0.5,1.1)	0.6 (0.4,0.9)	**0.022**
NLR, M (Q₁, Q₃)	4.4 (3.0,7.0)	5.3 (3.2,8.4)	5.8 (3.7,13.8)	**0.010**
ESR (mm/h), M (Q₁, Q₃)	26.0 (16.0,41.0)	31.0 (20.8,52.0)	44.0 (25.5,63.5)	**< 0.001**
CRP (mg/L), M (Q₁, Q₃)	3.30 (1.50,11.43)	5.03 (1.50,15.15)	12.10 (2.62,41.36)	**< 0.001**
C3 (g/L), M (Q₁, Q₃)	1.1 (1.0,1.2)	1.10 (1.0,1.2)	1.1 (0.9,1.2)	0.051
C4 (g/L), M (Q₁, Q₃)	0.3 (0.2,0.4)	0.3 (0.2,0.4)	0.3 (0.2,0.4)	0.599
CK (U/L), M (Q₁, Q₃)	68.0 (39.5,127.0)	64.0 (36.0,131.3)	69.0 (40.0,120.0)	0.885
LDH (U/L), M (Q₁, Q₃)	333.0 (276.5,445.0)	334.0 (289.8,410.8)	370.0 (303.5,477.5)	0.156
KL-6 (U/mL), M (Q₁, Q₃)	987 (622,1548)	973 (698,1559)	954 (639,1544)	0.627
SF (ng/mL), M (Q₁, Q₃)	664 (248,1184)	1004(522,2077)	1189(509,2486)	**< 0.001**
CD3^+^ (/µL), M (Q₁, Q₃)	528 (371,736)	486 (286,689)	394 (236,696)	**0.012**
CD3^+^ (%), M (Q₁, Q₃)	71.0 (60.1,79.5)	65.5 (54.6,74.0)	68.8 (58.8,78.9)	**0.008**
CD4^+^ (/µL), M (Q₁, Q₃)	339 (221,483)	287 (163,440)	232 (139,402)	**0.011**
CD4^+^ (%), Mean ± SD	42.7 ± 12.3	40.0 ± 13.2	41.6 ± 13.9	0.268
CD8^+^ (/µL), M (Q₁, Q₃)	173 (114,297)	144 (91,239)	116(65,196)	**< 0.001**
CD8^+^ (%), M (Q₁, Q₃)	24.0 (18.4,30.8)	21.5 (15.8,26.6)	21.0 (13.0,28.9)	**0.019**
CD4^+^/CD8^+^ ratio, M (Q₁, Q₃)	1.7 (1.3,2.5)	2.0 (1.3,2.8)	2.1 (1.4,3.2)	0.222

Anti-MDA5, anti-melanoma differentiation-associated protein-5 antibody; LYM, peripheral blood lymphocyte count; NLR, neutrophil-to-lymphocyte ratio; ESR, erythrocyte sedimentation rate; CRP, c-reactive protein; C3/4, complement 3/4; CK, creatine kinase; LDH, lactate dehydrogenase; KL-6, krebs von den lungen-6; SF, serum ferritin

A correlation analysis was performed between age and laboratory examinations (Supplementary Fig. S1). The analysis revealed positive correlations between age and several laboratory indicators: ESR (*r* = 0.218, *P* < 0.0001), CRP (*r* = 0.258, *P* < 0.0001), serum ferritin (*r* = 0.270, *P* < 0.0001), and NLR (*r* = 0.201, *P* < 0.001). Conversely, negative correlations with age were observed for lymphocyte count (*r *= -0.160, *P* = 0.004), CD3^+^ T cell count (*r *= -0.175, *P* = 0.002), CD4^+^ T cell count (*r *= -0.170, *P* = 0.002), CD8^+^ T cell count (*r *= -0.224, *P* < 0.0001), and CD8^+^ T cell ratio (*r *= -0.153, *P* = 0.006). No statistically significant correlations with age were found for B cells and NK cells (data not shown).

### Evaluation of mortality-associated factors across age groups

**Fig. 3:**
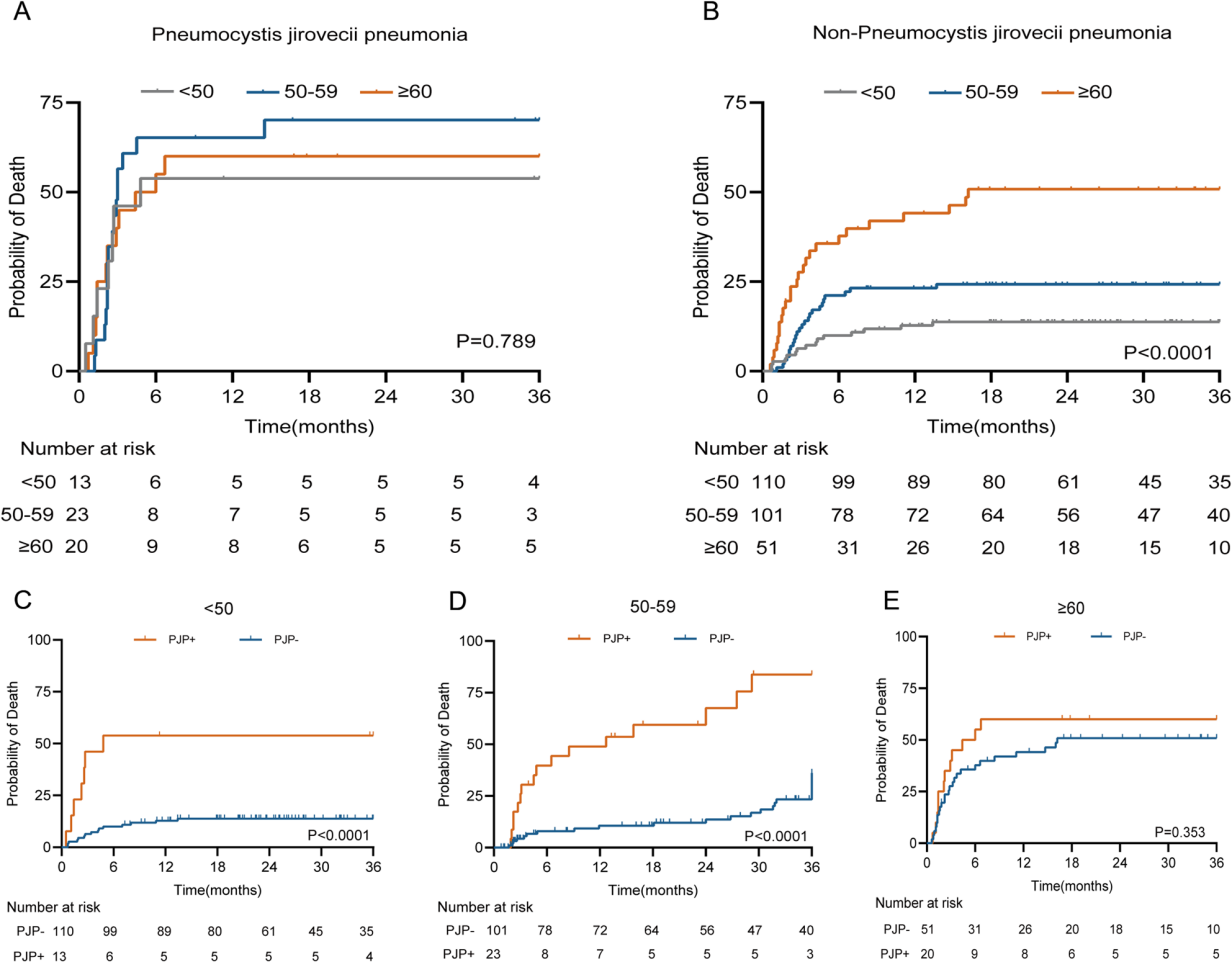
Survival analysis by infection status and age in anti-MDA5-positive dermatomyositis patients. Kaplan-Meier curves comparing survival across age groups: **(A)*** Pneumocystis jirovecii* pneumonia subset. **(B)** Non-*Pneumocystis jirovecii* pneumonia subset. (**C**) < 50-year-old subset by PJP status. (**D**) 50-59-year-old subset by PJP status. (**E**) ≥ 60-year-old subset by PJP status. Log-rank p-values shown for intergroup comparisons

To minimize the confounding impact of PJP, patients within each age group were stratified by PJP status. Kaplan-Meier survival analyses (Fig. 3) showed no significant difference in survival among the three age groups with PJP (*P* = 0.789) (Fig. 3A), while survival differed significantly among the three age groups without PJP (*P* < 0.0001) (Fig. 3B). Within the age groups, patients aged < 50 and 50–59 years demonstrated significantly worse survival if they had PJP compared to those without PJP (both *P* < 0.001) (Fig. 3C, D). However, in patients aged ≥ 60 years, survival did not differ significantly between those with and without PJP (*P* = 0.353) (Fig. 3E). These results indicate that both PJP infection and advanced age significantly impacted patient prognosis. In particular, among patients aged ≥ 60 years, survival did not significantly differ between those with and without PJP, suggesting that age had a more prominent influence on mortality in this group.

**Fig. 4:**
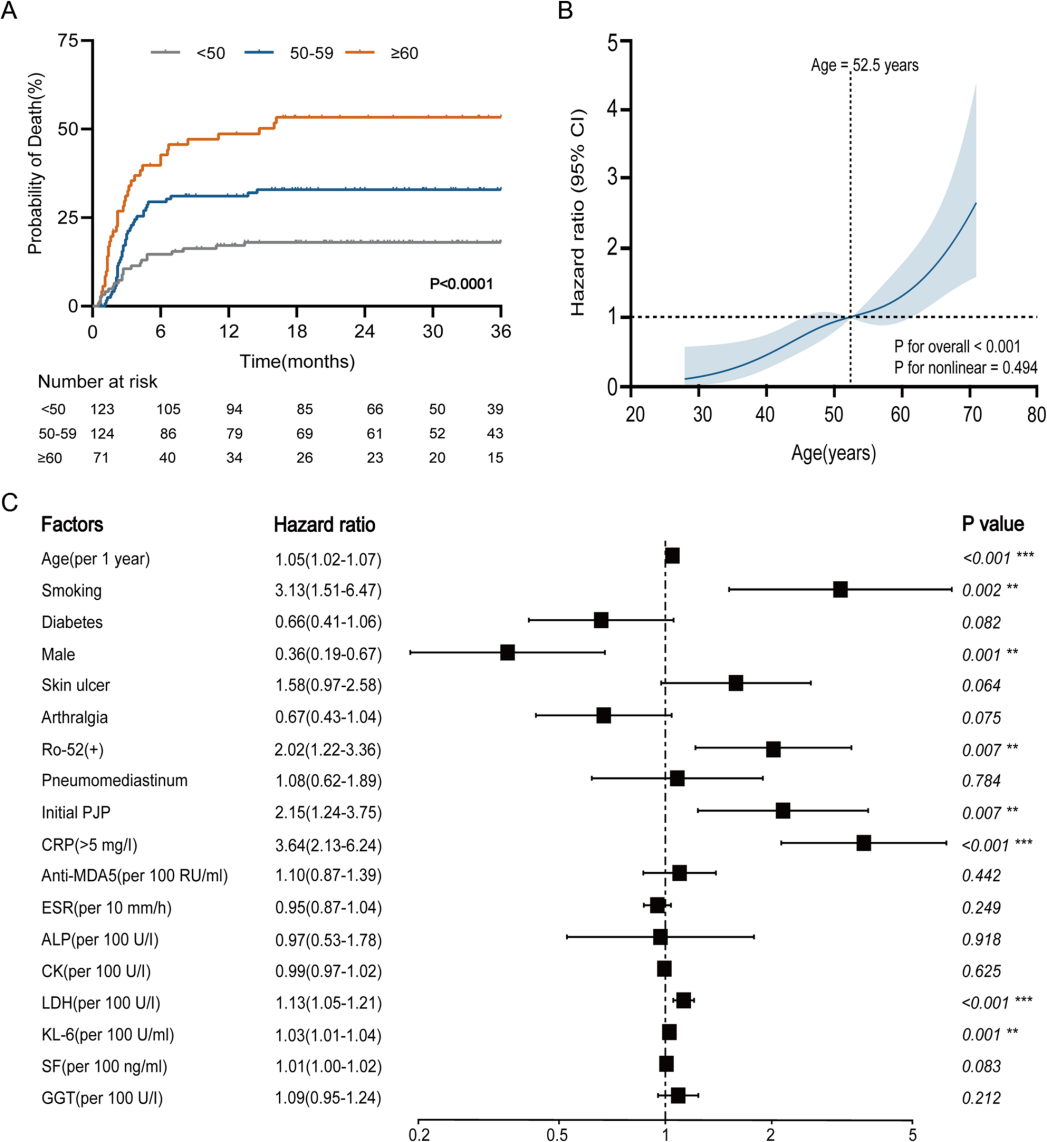
Prognosis and risk factor analysis in anti-MDA5-positive dermatomyositis across age groups. **(A)** Comparison of prognosis across age groups. **(B)** Association between age and 3-year mortality in patients. **(C)** Multivariate Cox regression analysis for identifying risk factors

Subsequently, a survival analysis of the entire cohort was conducted. Kaplan-Meier curves (Fig. 4A) demonstrated a significant difference in mortality rates among the three age groups (*P* < 0.001). To further characterize the relationship between age and mortality risk, a restricted cubic spline (RCS) analysis was performed (Fig. 4B), showing a significant linear association (*P* for overall < 0.001). The 3-year mortality risk increased gradually with age and rose sharply beyond 52.5 years.

To identify independent early predictors of mortality, both univariate and multivariate Cox regression analyses were performed. All variables included in the model, such as age, smoking history, anti-Ro-52 positivity, CRP, LDH, KL-6 levels, and the presence of PJP at the initial presentation, are parameters readily available during early hospitalization. The multivariate model (Fig. 4C) demonstrated that age, smoking history, anti-Ro-52 positivity, initial PJP, CRP > 5 mg/L, and elevated LDH and KL-6 levels were all independently associated with an increased risk of mortality.

Specifically, each 1-year increase in age was associated with a 5% increase in mortality risk (HR = 1.05, *P* < 0.001, 95% CI: 1.02–1.07). The smoking history was associated with a 213% higher mortality risk (HR = 3.13, *P* = 0.002, 95% CI: 1.51–6.47), and initial PJP conferred a 115% higher risk (HR = 2.15, *P* = 0.007, 95% CI: 1.24–3.75). Both Anti-Ro-52 positivity (HR = 2.02, *P* = 0.007, 95% CI: 1.22–3.36) and CRP > 5 mg/L (HR = 3.64, *P* < 0.001, 95% CI: 2.13–6.24) were also significantly associated with increased mortality risk.

### The nomogram developed based on patients with anti-MDA5+ DM

**Fig. 5:**
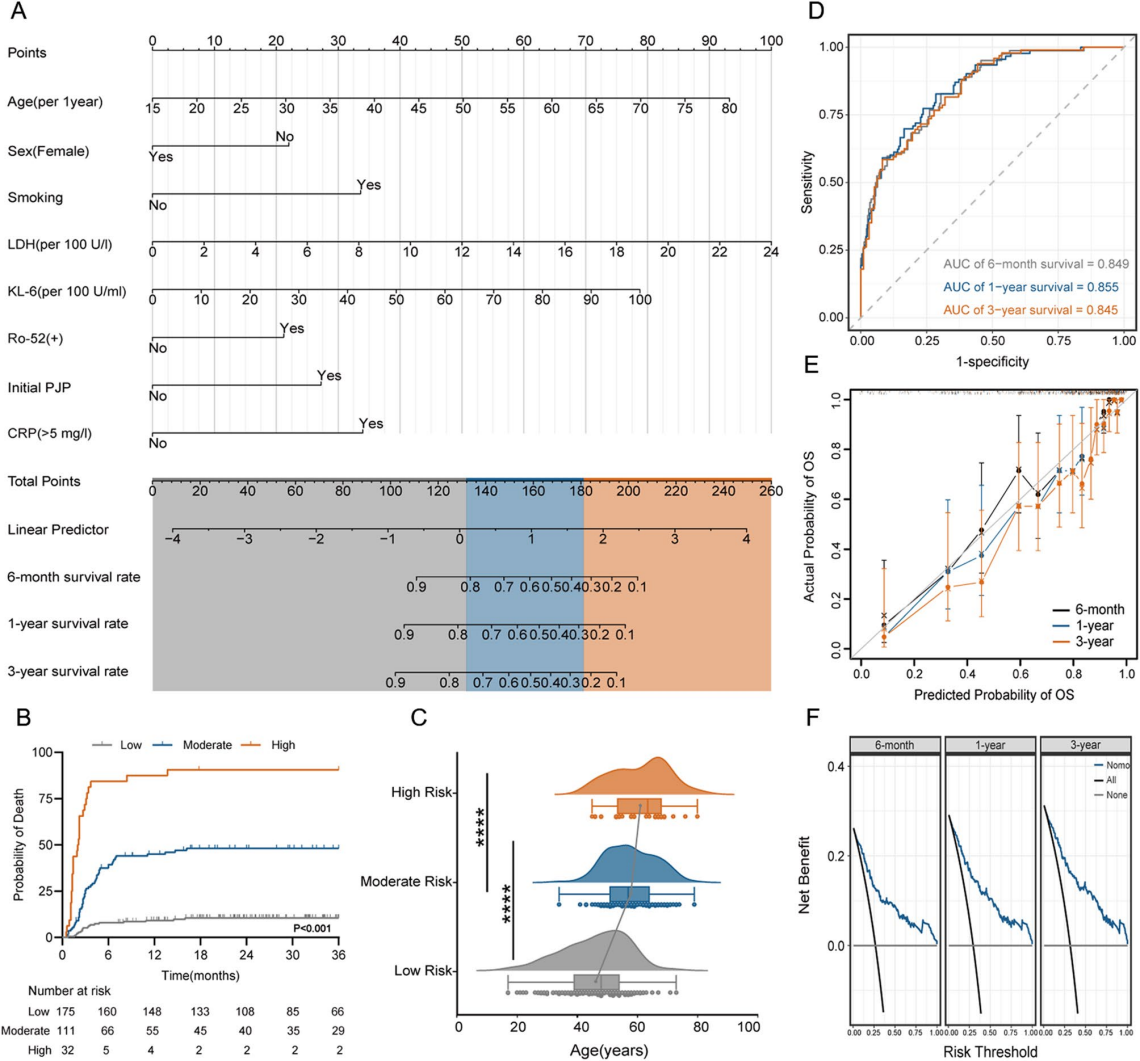
Nomogram for mortality prediction and prognostic evaluation in anti-MDA5-positive dermatomyositis. **(A)** Nomogram predicting mortality risk based on clinical variables. **(B)** Comparison of prognosis across risk groups. **(C)** Comparison of age distribution across different risk groups. **(D)** ROC curves of the disease mortality prediction model. **(E)** Calibration curves for 6-month, 1-year, and 3-year overall survival (OS) in anti-MDA5+ DM patients. **(F)** Decision curve analysis evaluating nomogram clinical utility. (*p < 0.05, **p < 0.01, ***p < 0.001, ****p < 0.0001)

Nomogram model (Fig. 5A) was constructed using eight variables identified as significant in the multivariate Cox regression analysis to predict the risk of disease-related mortality at 6 months, 1 year, and 3 years. The nomogram enables risk estimation by drawing vertical lines from the total score axis to the predicted outcome axis. Optimal cutoff values were determined using X-tile software (version 3.6.1), categorizing patients into low risk (175 individuals; total points ≤ 133), moderate risk (111 individuals; 133 < total points < 181), and high risk (32 individuals; total points ≥ 181) for overall survival (OS) prediction. Kaplan-Meier survival curves demonstrated excellent discrimination among the risk groups, with the high-risk group exhibiting a 3-year mortality rate of 90.6%, compared to 46.9% in the moderate-risk group and 10.3% in the low-risk group (Fig. 5B, *P* < 0.001). Furthermore, significant differences in age were observed between the moderate- and high-risk groups compared to the low-risk group (Fig. 5C, *P* < 0.001). The proportion of patients aged ≥ 60 years was 53.1% in the high-risk group, 37.8% in the moderate-risk group, and 6.9% in the low-risk group. Notably, patients in the high-risk group were significantly older than those in the low-risk group.

Additional analyses of demographic characteristics, clinical manifestations, and laboratory examinations across the risk groups are detailed in Supplementary Table S1-2. The c-index for the nomogram model was 0.811 (95% CI: 0.772–0.850), and the area under the ROC curve (AUC) for predicting survival at 6 months, 1 year, and 3 years was 0.849, 0.855, and 0.845, respectively (Fig. 5D). The calibration curves, based on 1000 bootstrap resamples, showed a strong agreement between predicted and observed risks of death at 6 months, 1 year, and 3 years (Fig. 5E). These results underscore the prognostic model’s robust discriminative ability for patient survival rates. Finally, decision curve analysis confirmed that the nomogram provides a net benefit for predicting disease-related mortality at 6 months, 1 year, and 3 years (Fig. 5F).

## Discussion

In this study, we identified significant age-related variations in clinical manifestations, comorbidities, laboratory examinations, and long-term mortality in a large cohort of Chinese patients with anti-MDA5+ DM. Patients aged ≥ 60 years exhibited a higher prevalence of cough, *Pneumocystis jirovecii* pneumonia (PJP), dyspnea, and rapidly progressive interstitial lung disease (RP-ILD), whereas rash and arthralgia were less common compared to those aged < 60 years. Age-related increases were also observed in alveolar-arterial oxygen gradient (AaDO₂), erythrocyte sedimentation rate (ESR), C-reactive protein (CRP), and serum ferritin levels, while PaO₂/FiO₂ ratios and lymphocyte counts declined with age.

In terms of treatment regimens, although no significant differences were observed in the use of dual or triple therapies across groups, patients aged ≥ 60 years used glucocorticoid monotherapy more frequently. To ensure this did not bias our prognostic analysis, we further examined this specific subgroup and found that the higher frequency of monotherapy was primarily due to an exceptionally rapid disease course in a small number of cases; notably, 10 out of the 13 elderly patients in this category died within just 5 days of admission (Supplementary Table S3). This indicates that the restricted therapy was a result of an extremely narrow clinical window rather than insufficient treatment intensity. Regarding prognosis, patients aged ≥ 60 years had the highest mortality rate, followed by those aged 50–59 years, with the best outcomes observed in those under 50. Our novel prognostic model, incorporating age and key risk factors, stratified patients into distinct risk categories. The high-risk group demonstrated significantly increased mortality and older age compared to low-risk patients.

Notably, the proportion of RP-ILD increased with age, and older patients were more prone to complications, particularly pulmonary infections such as PJP, which markedly impacted prognosis. Supporting this, a study by Yamaguchi et al. [18] found that patients over 60 years of age were more likely to develop RP-ILD and associated infections, leading to a poorer prognosis. Similarly, So et al. [25] identified age > 50 years as an independent risk factor for RP-ILD and developed the FLAW model to stratify this risk. In our study, analysis of arterial blood gas revealed that PaO₂/FiO₂ was negatively correlated with age, while AaDO₂ was positively correlated with age, suggesting a decline in oxygenation capacity among older patients. These findings further support the notion that aging is associated with greater susceptibility to RP-ILD and pulmonary infections.

We further distinguished between initial (i-) and post-treatment (pt-) PJP events and evaluated their cumulative incidence. The incidence of PJP increased with age in anti-MDA5+ DM patients. PJP was associated with a significantly higher risk of mortality. Even within the same age group, patients with PJP had worse outcomes, except in those aged ≥ 60 years, where age appeared to exert a stronger influence on prognosis than PJP status. These findings emphasize the need for heightened infection surveillance and early intervention in older patients. Consistent with our findings, Chen et al. reported that most PJP cases in anti-MDA5+ DM occurred within the first 3 months and were associated with high early mortality [26]. Yang et al. further identified older age as a risk factor for PJP, with a 30-day mortality exceeding 50% among PJP patients [27]. Liu et al. demonstrated that prophylactic use of trimethoprim-sulfamethoxazole (TMP-SMX) significantly reduced both PJP incidence and all-cause mortality in newly diagnosed anti-MDA5+ DM patients [28]. To address potential bias, we further evaluated whether the implementation of PJP prophylaxis differed among age groups. While prophylaxis rates varied in the full cohort, our analysis of a restricted cohort, which excluded patients with initial PJP and those with early death, showed no significant difference in the proportion of patients receiving prophylaxis across age groups (Supplementary Table S4). Taken together, these findings identify PJP and advanced age as key factors contributing to poor prognosis in anti-MDA5+ DM. By early infection surveillance, we emphasize early detection of PJP for patients suspicious of PJP infection rather than routine screening of asymptomatic patients. Prophylactic antimicrobial strategies are also essential, particularly in older patients, where close monitoring of respiratory function and prompt, aggressive treatment are warranted even in the absence of overt symptoms [26, 29, 30].

Laboratory examinations further revealed age-related elevations in ESR, CRP, and serum ferritin. We also observed a progressive decline in peripheral lymphocyte counts with age, with significantly lower levels in those aged ≥ 60 years. Lu and Peng et al. [31] previously noted that elevated serum ferritin and reduced peripheral lymphocyte counts are associated with poor outcomes. A large cohort study of anti-MDA5+ DM patients in China [32] reported that those with the lowest peripheral lymphocyte counts had a high incidence of RP-ILD and a poor prognosis. Furthermore, detailed analyses [33] have shown that reductions in peripheral CD3^+^ T cells, including CD4^+^ and CD8^+^ subsets, correlate with disease severity and adverse outcomes. Additionally, a meta-analysis [34] further linked aging with increased levels of inflammatory markers such as IL-6 and CRP. Our results corroborate these findings, showing elevated ESR, CRP, and serum ferritin levels, along with significant declines in CD3^+^ T cells (including CD4^+^ and CD8^+^ subsets) in older patients, reinforcing their association with poor prognosis.

Building upon previous research, which identified advanced age, anti-Ro-52 antibody positivity, and elevated CRP, KL-6, and LDH levels as predictors of adverse outcomes in anti-MDA5+ DM [17, 35, 36]. Our study extends these findings by revealing additional independent risk factors for poor prognosis in anti-MDA5+ DM patients through multifactorial Cox regression analysis. Specifically, initial PJP and smoking history were identified as independent risk factors. Consistent with previous studies [18, 19], we found that age ≥ 60 years is an independent predictor of poor prognosis in anti-MDA5+ DM patients. Moreover, prior research indicates that the poor prognosis of anti-MDA5+ DM is partially attributable to a high prevalence of infections and lymphopenia, which increases susceptibility to opportunistic infections [37, 38]. DM/PM patients are particularly prone to opportunistic infections, with PJP being notably prevalent and associated with high mortality in anti-MDA5+ DM patients [26, 39]. A retrospective Chinese study further demonstrated that patients with invasive pulmonary aspergillosis (IPA), RP-ILD, or co-infection with PJP, along with reduced lymphocyte counts, had significantly worse outcomes [40].

With respect to mortality differences across age and sex, our study found that the prevalence of anti-MDA5+ DM was higher in females across all age groups, consistent with previous reports from China and other countries [41–43]. However, among patients aged ≥ 50 years, males exhibited higher mortality rates than females. In China, smoking remains predominantly prevalent among males, largely due to cultural norms. A multicenter registry of patients with IIM showed an association between smoking and interstitial lung disease [44]. Liu et al. [45] recently confirmed smoking as an independent risk factor for mortality in patients with anti-MDA5+ DM-ILD. Similarly, Montes et al. [46] found that smoking exposure contributes to chronic cumulative damage in patients with SLE. Large multicenter studies are needed to further explore the role of smoking in chronic pulmonary injury and prognosis in anti-MDA5+ DM.

The CRAFT model [47] (C-reactive protein-to-albumin ratio, red blood cell distribution width-coefficient of variation, fever status, and CD3^+^ T cells) and the CROSS model [48] (C-reactive protein levels, anti-Ro-52 antibody positivity, short disease duration, and male sex) have been developed by Chinese researchers to predict the risk of RP-ILD in patients with anti-MDA5+ DM. To evaluate long-term outcomes in patients with anti-MDA5+ DM, we developed a prognostic nomogram incorporating age and PJP infection, two factors strongly associated with increased mortality and poorer prognosis. While advanced age reflects the baseline vulnerability of patients, PJP infection highlights the importance of early detection. Patients were stratified into high-, moderate-, and low-risk groups. The high-risk group tended to include older patients and those with a higher incidence of PJP, reflecting a relatively worse prognosis. This risk stratification provides a practical tool for early identification of patients at greater risk, enabling more personalized and timely management. Risk-based stratification and individualized treatment remain important for improving long-term outcomes in anti-MDA5+ DM. For patients at high risk, early and intensified therapeutic interventions are warranted to improve survival, mitigate complications, and enhance long-term quality of life.

### Limitation

This study has several limitations. First, as a single-center retrospective study, the findings may not be fully generalizable. Second, the cohort may be biased toward more severe cases because most newly diagnosed or active patients were hospitalized, while milder outpatient cases were underrepresented. Third, although known mortality-related factors were adjusted for, residual treatment-related confounding cannot be fully excluded. Fourth, standardized high-resolution CT (HRCT) protocols were lacking, limiting quantitative assessment of ILD severity. Finally, the prognostic nomogram was only internally validated, and external validation is required before clinical application. Large-scale, prospective, multicenter studies are needed to further confirm the model’s performance and generalizability.

## Conclusion

Younger patients with anti-MDA5+ DM are more likely to present with rash and arthralgia, whereas older patients exhibit a poorer prognosis characterized by higher rates of cough, PJP, dyspnea, RP-ILD, elevated inflammatory markers, and reduced T lymphocyte counts. The proposed nomogram effectively stratifies patients into distinct risk groups and underscores the prognostic significance of both advanced age and PJP infection, providing a practical tool for early identification and management of high-risk individuals.

## Supplementary Information

Below is the link to the electronic supplementary material.

Supplementary Material 1

Supplementary Material 2

Supplementary Material 3

Supplementary Material 4

Supplementary Material 5

## Data Availability

The data is available in this article, and further inquiries can be directed to the corresponding authors.

## Abbreviations

Anit-MDA5+ DM: Anti-melanoma differentiation-associated gene 5 antibody-positive dermatomyositis
AaDO₂: Alveolar-arterial oxygen gradient
CNI: Calcineurin inhibitor
CRP: C-reactive protein
CYC: Cyclophosphamide
DLCO: Diffusing capacity of the lungs for carbon monoxide
ESR: Erythrocyte sedimentation rate
FVC: Forced vital capacity
GC: Glucocorticoids
HRCT: High-resolution CT
IIM: Idiopathic inflammatory myopathy
IPA: Invasive pulmonary aspergillosis
IVIG: Intravenous immunoglobulin
LORA: Late-onset RA
NLR: Neutrophil-to-lymphocyte ratio
NPSLE: Neuropsychiatric systemic lupus erythematosus
PJP: *Pneumocystis jirovecii* pneumonia
RA: Rheumatoid arthritis
RP-ILD: Rapidly progressive interstitial lung disease
SF: Serum ferritin
SLE: Systemic lupus erythematosus
SS: Sjögren’s syndrome
