# Supplementary material for: Clinical characteristics and long-term prognosis of anti-MDA5-positive dermatomyositis: a comparative study across age groups
Source: Orphanet J Rare Dis. 2026 Apr 11;21:211. doi: 10.1186/s13023-026-04345-y (PMC13224566; doi:10.1186/s13023-026-04345-y)
Supplement: Supplementary file 2 — Supplementary Material 2 [file 13023_2026_4345_MOESM2_ESM.docx]

**Table S1: Baseline demographic manifestations, clinical features, treatments, and outcome across risk groups.**

| Variables | Low (n = 175) | Moderate (n = 111) | High (n = 32) | *P* |
| --- | --- | --- | --- | --- |
|  |  |  |  |  |
| **Demographic features** | | | | |
| Age(years), M (Q₁, Q₃) | 48.0 (39.0,54.0) | 57.0 (51.0,64.0) | 63.5 (53.8,68.0) | **<.001** |
| Male, n (%) | 71 (40.57) | 27 (24.32) | 17 (53.12) | **0.002** |
| Disease duration (months), M (Q₁, Q₃) | 2.17 (1.2,3.9) | 1.5 (1.0,2.9) | 1.2 (1.0,2.1) | **<.001** |
| Smoking, n (%) | 15 (8.6) | 9 (8.1) | 13 (40.6) | **<.001** |
| Diabetes, n (%) | 45 (25.7) | 35 (31.5) | 11 (34.4) | 0.427 |
| **Clinical features** | | | | |
| ILD, n (%) | 161 (92.0) | 109 (98.2) | 32 (100.0) | **0.025** |
| RP-ILD, n (%) | 55 (31.4) | 77 (69.4) | 30 (93.8) | **<.001** |
| Rash, n (%) | 161 (92.0) | 97 (87.4) | 25 (78.1) | 0.056 |
| Skin ulcer, n (%) | 24 (13.7) | 24 (21.6) | 10 (31.3) | **0.032** |
| Mechanic's hands, n (%) | 39 (22.3) | 23 (20.7) | 8 (25.0) | 0.869 |
| Heliotrope sign, n (%) | 118 (67.4) | 61 (55.0) | 13 (40.6) | **0.006** |
| Gottron’s sign, n (%) | 94 (53.7) | 58 (52.3) | 12 (37.5) | 0.237 |
| Myalgia, n (%) | 65 (37.1) | 33 (29.7) | 7 (21.9) | 0.158 |
| Muscle fatigability, n (%) | 68 (38.9) | 41 (36.9) | 6 (18.8) | 0.092 |
| Cough, n (%) | 115 (65.7) | 86 (77.5) | 26 (81.3) | **0.043** |
| Chest tightness, n (%) | 99 (56.6) | 83 (74.8) | 31 (96.9) | **<.001** |
| Dyspnea, n (%) | 52 (29.7) | 55 (49.6) | 28 (87.5) | **<.001** |
| Arthralgia, n (%) | 89 (50.9) | 48 (43.2) | 11 (34.4) | 0.157 |
| **Complications** | | | | |
| Pneumomediastinum, n (%) | 9 (5.1) | 16 (14.4) | 6 (18.8) | **0.007** |
| Pneumocystis jirovecii pneumonia, n (%) | 13 (7.4) | 26 (23.4) | 17 (53.1) | **<.001** |
| i-PJP, n (%) | 2 (1.1) | 13 (11.7) | 17 (53.1) | **<.001** |
| pt-PJP, n (%) | 11 (6.3) | 13 (11.7) | 0 (0.0) | 0.056 |
| **Pulmonary function^1^** | | | | |
| FVC%, M (Q₁, Q₃) | 77.0 (63.3,87.1) | 62.4 (56.7,79.6) | NA | **<.001** |
| FVC, L, M (Q₁, Q₃) | 2.4 (2.0,3.1) | 2.1 (1.8,2.4) | NA | **<.001** |
| DLCO, ml/min/mmHg, M (Q₁, Q₃) | 4.7 (3.7,5.8) | 3.7 (3.0,4.8) | NA | **<.001** |
| DLCO%, M (Q₁, Q₃) | 55.9 (48.3,68.3) | 50.2 (35.6,57.6) | NA | **<.001** |
| **Arterial blood gas test** | | | | |
| PaO_2_/FiO_2_(mmHg), M (Q₁, Q₃) | 322.8 (250.9,375.0) | 230.0 (159.2,314.8) | 123.0 (94.0,182.3) | **<.001** |
| AaDO_2_(mmHg), M (Q₁, Q₃) | 35.2 (20.8,92.1) | 100.8 (36.9,169.6) | 209.7 (133.0,409.4) | **<.001** |
| **Treatment regimens** | | | | |
| Dosage of MP (mg/d), M (Q₁, Q₃) | 80.0 (80.0,80.0) | 80.0 (80.0,80.0) | 80.0 (80.0,80.0) | 0.961 |
| GC mono, n (%) | 10 (5.7) | 10 (9.0) | 15 (46.9) | **<.001** |
| **Dual therapy, n (%)** | | | | |
| MP+CNI, n (%) | 45 (25.7) | 31 (27.9) | 5 (15.6) | 0.369 |
| MP+CYC, n (%) | 21 (12.0) | 19 (17.1) | 4 (12.5) | 0.462 |
| MP+JAKi, n (%) | 11 (6.3) | 4 (3.6) | 1 (3.1) | 0.524 |
| **Triple therapy^2^, n (%)** | | | | |
| MP+CNI+CYC, n (%) | 50 (28.6) | 31 (27.9) | 4 (12.5) | 0.158 |
| MP+CNI+JAKi, n (%) | 36 (20.6) | 19 (17.1) | 2 (6.3) | 0.146 |
| **Add-on therapy, n (%)** | | | | |
| RTX, n (%) | 1 (0.6) | 1 (0.9) | 0 (0.0) | 1.000 |
| IVIG, n (%) | 101 (57.7) | 91 (82.0) | 23 (71.9) | **<.001** |
| **Outcome** | | | | |
| Follow-up duration | 29.1 (18.3,36.0) | 11.33 (3.0,36.0) | 2.07 (1.3,3.1) | **<.001** |
| Deaths within 3 years, n (%) | 18 (10.3) | 52 (46.9) | 29 (90.6) | **<.001** |
| Female, n (%) | 10 (9.6) | 39 (46.4) | 13 (86.7) | **<.001** |
| Male, n (%) | 8 (11.3) | 13 (48.2) | 16 (94.1) | **<.001** |

Disease duration, the time from symptom onset to diagnosis; ILD, interstitial lung disease; RP-ILD, rapidly progressive interstitial lung disease; PJP, pneumocystis jirovecii pneumonia; i-PJP, initial PJP; pt-PJP, post-treatment PJP; FVC, forced vital capacity; DLCO, diffusing capacity for carbon monoxide; AaDO_2_, arterial-alveolar oxygen gradient, P(A-a)O_2_=(PiO_2_-PaCO_2_/R)-PaO_2_; GC mono, only glucocorticoids were administered initially; MP, methylprednisolone; CNI, calcineurin inhibitors; CYC, cyclophosphamide; JAKi, janus kinase inhibitors; RTX, rituximab; IVIG, intravenous immunoglobulin.
^1^Pulmonary function tests were often missing in patients ≥60 y due to RP-ILD, test intolerance, and risk of further lung injury.

^2^Triple therapy refers to glucocorticoids combined with any two immunosuppressants. In our cohort, the most commonly employed regimens were MP + CNI + CYC or MP + CNI + JAKi.
