# Supplementary material for: Clinical characteristics and long-term prognosis of anti-MDA5-positive dermatomyositis: a comparative study across age groups
Source: Orphanet J Rare Dis. 2026 Apr 11;21:211. doi: 10.1186/s13023-026-04345-y (PMC13224566; doi:10.1186/s13023-026-04345-y)
Supplement: Supplementary file 3 — Supplementary Material 3 [file 13023_2026_4345_MOESM3_ESM.docx]

**Table S2: Baseline laboratory examinations across risk groups.**

| Variables | Low (n = 175) | Middle (n = 111) | High (n = 32) | *P* |
| --- | --- | --- | --- | --- |
|  |  |  |  |  |
| Anti-Ro52(+), n (%) | 76 (43.4) | 84 (75.7) | 27 (84.4) | **<.001** |
| Anti-MDA5 (U/mL), M (Q₁, Q₃) | 176.3 (152.1,199.6) | 179.2 (156.5,205.4) | 172.3 (140.5,205.6) | 0.554 |
| LYM (10^9/L)，M (Q₁, Q₃) | 0.85 (0.60,1.14) | 0.67 (0.44,0.88) | 0.49 (0.42,0.62) | **<.001** |
| NLR, M (Q₁, Q₃) | 3.9 (2.7,6.3) | 5.8 (3.8,9.9) | 12.5 (9.2,18.7) | **<.001** |
| ESR (mm/h), M (Q₁, Q₃) | 24.0 (12.0,37.5) | 43.0 (25.5,60.5) | 50.0 (35.3,66.8) | **<.001** |
| CRP (mg/L), M (Q₁, Q₃) | 1.5 (1.3,3.9) | 12.1 (5.8,29.9) | 34.3 (11.5,61.3) | **<.001** |
| C3 (g/L), M (Q₁, Q₃) | 1.1 (0.9,1.2) | 1.1 (0.9,1.2) | 1.1 (0.9,1.2) | 0.702 |
| C4 (g/L), M (Q₁, Q₃) | 0.3 (0.3,0.4) | 0.3 (0.2,0.4) | 0.3 (0.3,0.4) | 0.188 |
| CK (U/L), M (Q₁, Q₃) | 64.0 (39.0,120.5) | 74.0 (35.5,134.0) | 70.0 (37.8,135.3) | 0.967 |
| LDH (U/L), M (Q₁, Q₃) | 315.0 (266.0,391.5) | 366.0 (297.0,444.0) | 581.5 (420.5,839.0) | **<.001** |
| KL-6 (U/mL), M (Q₁, Q₃) | 837.0 (601.0,1284.5) | 1014.0 (704.0,1739.0) | 1445.0 (941.8,2567.3) | **<.001** |
| SF (ng/mL), M (Q₁, Q₃) | 687.0 (287.5,1237.2) | 1188.7 (551.7,2124.0) | 1603.8 (751.4,3379.0) | **<.001** |
| CD3+ (/μL), M (Q₁, Q₃) | 570.0 (407.0,775.0) | 398.0 (243.5,648.5) | 325.6 (177.3,523.5) | **<.001** |
| CD3+ (%), M (Q₁, Q₃) | 68.8 (58.9,78.3) | 68.1 (59.1,76.0) | 69.9 (52.2,75.9) | 0.499 |
| CD4+ (/μL), M (Q₁, Q₃) | 358.0 (229.0,483.9) | 237.0 (137.0,396.5) | 197.0 (96.1,269.7) | **<.001** |
| CD4+ (%), Mean ± SD | 41.9 ± 12.3 | 41.4 ± 13.1 | 39.1 ± 16.5 | 0.545 |
| CD8+ (/μL), M (Q₁, Q₃) | 187.0 (122.8,292.0) | 116.0 (73.5,193.0) | 100.7 (63.0,180.0) | **<.001** |
| CD8+ (%), Mean ± SD | 24.3 ± 9.5 | 22.3 ± 11.2 | 23.7 ± 14.7 | 0.301 |
| CD16+CD56+ (/μL), M (Q₁, Q₃) | 78.0 (44.4,138.00) | 61.3 (26.4,100.0) | 36.2 (25.6,51.1) | **<.001** |
| CD16+CD56+ (%), M (Q₁, Q₃) | 9.6 (5.5,15.99) | 9.7 (5.3,16.0) | 6.5 (4.2,11.0) | 0.072 |
| CD19+ (/μL), M (Q₁, Q₃) | 117.0 (73.5,239.18) | 113.0 (58.3,169.5) | 82.9 (30.5,136.3) | **0.004** |
| CD19+ (%), M (Q₁, Q₃) | 15.6 (9.5,25.08) | 16.5 (12.1,24.6) | 14.7 (12.2,22.7) | 0.554 |
| Th/Ts, M (Q₁, Q₃) | 1.9 (1.3,2.55) | 1.9 (1.4,3.3) | 1.9 (1.3,2.7) | 0.403 |

Anti-MDA5, anti-melanoma differentiation-associated protein-5 antibody; LYM, peripheral blood lymphocyte count; NLR, neutrophil-to-lymphocyte ratio; ESR, erythrocyte sedimentation rate; CRP, c-reactive protein; C3/4, complement 3/4; CK, creatine kinase; LDH, lactate dehydrogenase; KL-6, krebs von den lungen-6; SF, serum ferritin.
