# Supplementary material for: Clinical characteristics and long-term prognosis of anti-MDA5-positive dermatomyositis: a comparative study across age groups
Source: Orphanet J Rare Dis. 2026 Apr 11;21:211. doi: 10.1186/s13023-026-04345-y (PMC13224566; doi:10.1186/s13023-026-04345-y)
Supplement: Supplementary file 4 — Supplementary Material 4 [file 13023_2026_4345_MOESM4_ESM.docx]

**Table S3: Clinical Course of Patients Aged** ≥**60 Years Receiving Glucocorticoid Monotherapy.**

| Patient ID | Gender | Age at diagnosis(years) | Disease duration(months) | Anti-MDA5 antibody titer(U/ml) | Time from diagnosis to death(days) |
| --- | --- | --- | --- | --- | --- |
| PA1 | Male | 63 | 3.1 | 183.5 | 1 |
| PA2 | Male | 63 | 5.9 | 181.3 | 1 |
| PA3 | Female | 64 | 2.2 | 169.9 | 4 |
| PA4 | Female | 66 | 1.1 | 203.4 | 1 |
| PA5 | Male | 66 | 2.2 | 216.3 | 2 |
| PA6 | Male | 67 | 0.9 | 119.2 | 2 |
| PA7 | Male | 67 | 1.3 | 224.64 | 1 |
| PA8 | Female | 69 | 1.1 | 186.9 | 45 |
| PA9 | Female | 69 | 1.4 | 205.8 | 2 |
| PA10 | Male | 69 | 1.4 | 205.5 | 3 |
| PA11 | Female | 71 | 4.2 | 76.9 | 4 |
| PA12 | Female | 76 | 1.3 | 225.6 | 99 |
| PA13 | Female | 80 | 3.2 | 128.2 | 155 |
