# Supplementary material for: Clinical characteristics and long-term prognosis of anti-MDA5-positive dermatomyositis: a comparative study across age groups
Source: Orphanet J Rare Dis. 2026 Apr 11;21:211. doi: 10.1186/s13023-026-04345-y (PMC13224566; doi:10.1186/s13023-026-04345-y)
Supplement: Supplementary file 5 — Supplementary Material 5 [file 13023_2026_4345_MOESM5_ESM.docx]

**Table S4: Distribution of PJP Prophylaxis Across Age Groups.**

| Cohort | Age group | n | Prophylactic TMP-SMX, n (%) | No prophylaxis, n (%) | P value |
| --- | --- | --- | --- | --- | --- |
| Full cohort | <50 | 123 | 66（53.7） | 57（46.3） | 0.023 |
|  | 50-59 | 124 | 62（50.0） | 62（50.0） |  |
|  | ≥60 | 71 | 24（33.8） | 47（66.2） |  |
| Restricted cohort^*^ | <50 | 107 | 66（61.7） | 41（38.3） | 0.480 |
|  | 50-59 | 100 | 62（62.0） | 38（38.0） |  |
|  | ≥60 | 46 | 24（52.2） | 22（47.3） |  |

^*^Restricted cohort excludes patients with initial PJP or who died during the initial admission.
